# Association of Asymmetric and Symmetric Dimethylarginine with Inflammation in the Population-Based Study of Health in Pomerania

**DOI:** 10.3390/biom13111612

**Published:** 2023-11-04

**Authors:** Martin Sebastian Winkler, Martin Bahls, Rainer H. Böger, Till Ittermann, Marcus Dörr, Nele Friedrich, Edzard Schwedhelm

**Affiliations:** 1 Department of Anesthesiology, Emergency and Intensive Care Medicine, University of Göttingen, 37075 Göttingen, Germany; 2 Department of Internal Medicine B, University Medicine Greifswald, 17475 Greifswald, Germany; 3 German Center for Cardiovascular Research (DZHK), Partner Site Greifswald, 17475 Greifswald, Germany; 4 Institute of Clinical Pharmacology and Toxicology, University Medical Centre Hamburg-Eppendorf, 20246 Hamburg, Germanyschwedhelm@uke.de (E.S.); 5 Institute for Community Medicine, University Medicine Greifswald, 17475 Greifswald, Germany; 6 Institute of Clinical Chemistry and Laboratory Medicine, University Medicine Greifswald, 17475 Greifswald, Germany; 7 German Center for Cardiovascular Research (DZHK), Partner Site Hamburg/Kiel/Lübeck, 20246 Hamburg, Germany

**Keywords:** a proliferation-inducing ligand, asymmetric dimethylarginine, inflammation, soluble cluster of differentiation 30, Study of Health in Pomerania, symmetric dimethylarginine, tumor necrosis factor receptor

## Abstract

The amino acids arginine (Arg), asymmetric (ADMA) and symmetric dimethylarginine (SDMA) are related to nitric oxide (NO) metabolism and potential markers of two different disease entities: cardiovascular disease such as atherosclerosis and systemic inflammation in critically ill patients with sepsis. Although very different in their pathophysiological genesis, both entities involve the functional integrity of blood vessels. In this context, large population-based data associating NO metabolites with proinflammatory markers, e.g., white blood cell count (WBC), high-sensitivity C-reactive protein (hsCRP), and fibrinogen, or cytokines are sparse. We investigated the association of Arg, ADMA and SDMA with WBC, hsCRP, and fibrinogen in 3556 participants of the Study of Health in Pomerania (SHIP)-TREND study. Furthermore, in a subcohort of 456 subjects, 31 inflammatory markers and cytokines were analyzed. We identified Arg and SDMA to be positively associated with hsCRP (β coefficient 0.010, standard error (SE) 0.002 and 0.298, 0.137, respectively) as well as fibrinogen (β 5.23 × 10^−3^, SE 4.75 × 10^−4^ and 0.083, 0.031, respectively). ADMA was not associated with WBC, hsCRP, or fibrinogen. Furthermore, in the subcohort, Arg was inversely related to a proliferation-inducing ligand (APRIL). SDMA was positively associated with osteocalcin, tumor necrosis factor receptor 1 and 2, and soluble cluster of differentiation 30. Our findings provide new insights into the involvement of Arg, ADMA, and SDMA in subclinical inflammation in the general population.

## 1. Introduction

Nitric oxide (NO)-regulated processes are universal and involved in many forms of systemic chronic, acute and sterile, but also pathogen-related, inflammatory responses such as atherosclerosis or complicated infections with signs of blood pressure and immune dysregulation and septic shock. Since the systemic NO level is considered as a marker for the degree of inflammation, it cannot be directly measured due to the extremely short half-life of seconds. Therefore, one may indirectly evaluate NO synthase (NOS) activity by the substrate arginine (Arg) by levels and the balance of the direct NOS inhibitor asymmetric dimethylarginin (ADMA) and the Arg transport inhibitor symmetric dimethylarginin (SDMA). There is strong evidence that alterations in the Arg:ADMA or Arg:SDMA ratio are surrogate markers for the severity of blood pressure dysregulation in shock states observed in patients treated in intensive care units (ICU) [1,2,3].

However, markers which allow prediction of survival or morbidity in critical care, when homeostasis is severely affected and compromised, often fail in preclinical settings due to low sensitivity and specificity in early and “pre-critical” states. This is obvious in infectious disease, with a dysregulated host response leading to organ failure defined as sepsis. Screening tools for patients have been discussed for decades and, again, the latest sepsis guidelines only recommend clinical signs such as the respiratory rate, neural status and systolic BP, which are consolidated in the quick sepsis-related organ failure assessment score (qSOFA) [4,5]. Interestingly, no laboratory marker in the emergency or outpatient setting is recommended or even established to identify patients at risk. The current focus and understanding of sepsis, which is an immunological response, should motivate us to identify biomarkers, which are better reflecting a process rather than describing a clinical status. Nevertheless, general practitioners still use traditional markers such as white blood cell count (WBC), C-reactive protein (CRP) or fibrinogen to identify an apparent infection or the severity of systemic invasion. That such a marker-based screening is useful in chronic systemic inflammatory disease has been shown for high-sensitivity CRP (hsCRP) and cardiovascular risk [6]. However, no such marker exists for the most common causes of death, which are still infectious diseases triggering septic responses [7].

Arg, ADMA and SDMA are markers for sepsis severity and related to traditional markers in the ICU setting [3,8,9,10]. However, no data exist on whether inflammatory markers (e.g., WBC, hsCRP and fibrinogen) are associated with Arg, ADMA and SDMA in a population-based setting, excluding individuals taking anti-inflammatory and anti-rheumatic drugs typical for chronic inflammatory disease treatment. We previously defined reference intervals for ADMA and SDMA in the Study of Health in Pomerania (SHIP)-START study [11]. Moreover, in the SHIP-TREND cohort, we investigated the inflammatory marker sphingosine-1-phosphate (S1P), which was found to be related to atherosclerosis and periodontitis [12,13]. Finally, in a subcohort of 469 study participants of the SHIP-TREND cohort, we measured the plasma concentration of cytokines, which provided new insights into the impact of hepatic and pancreatic fat on systemic inflammation [14].

Therefore, in this study, we aim to investigate the associations between surrogate markers for NO metabolism, Arg, ADMA and SDMA, and inflammatory markers or cytokines.

## 2. Materials and Methods

### 2.1. Study Population

All SHIP participants are inhabitants of West Pomerania, a rural area nestled in the northeast of Germany. Building upon the original SHIP-START cohort, SHIP-TREND was set up as a second, distinct population-based cohort. Extensive documentation on SHIP-TREND is available elsewhere [15,16]. Out of 8826 invited inhabitants of West Pomerania handpicked at random, 4420 individuals (50.1%), 2145 men and 2275 women responded and underwent medical examinations between 2008 and 2012 [17]. The SHIP-TREND study protocol was approved by the Ethics Committee of the University Medicine Greifswald, Germany (approval BB 39/08). SHIP-TREND was conducted following the principles of the Declaration of Helsinki and informed written consent was obtained from all 4420 study participants. Out of these eligible candidates, 317 subjects were excluded due to missing values for Arg, ADMA, SDMA or classical inflammatory markers (Figure S1). Further exclusion criteria were history of cancer, an estimated glomerular filtration rate (eGFR) below 30 mL/min/1.73 m^2^, a left ventricular ejection fraction smaller than 40 %, intake of stomatological preparations (Anatomical Therapeutic Chemical (ATC) code: A01), antidiarrheals, intestinal anti-inflammatory/anti-infective agents (A07), 3-hydroxy-3-methyl-glutaryl-coenzyme A (HMG-CoA) reductase inhibitors (C10AA), corticosteroids, dermatological preparations (D07), corticosteroids for systemic use (H02), anti-inflammatory and antirheumatic products (M01) or glucocorticoids (R05BA), or missing data in the confounders. The final study population regarding analyses for hsCRP, WBC or fibrinogen comprised 3556 individuals. Furthermore, in a subsample of 456 SHIP-TREND participants, cytokine measurements were performed and available for analyses (see Section 2.4. cytokine measurements).

### 2.2. General Characteristics of the Study Population

Each participant enrolled in the SHIP-TREND study underwent a series of standardized medical examinations, including blood sampling, and an extensive computer-aided personal interview. Information on sociodemographic characteristics and medical histories was collected. Participant’s smoking status, categorized as current smoker or non-smoker, was self-reported. Standardized measurements of body weight and height were obtained with calibrated scales during physical examinations. The body mass index (BMI) was calculated by dividing the weight by the square of the height (kg/m^2^). Waist circumference was measured with an inelastic tape, accurate to the nearest 0.1 cm. The measurement was taken at the midpoint between the lower rib margin and the iliac crest in a horizontal plane, while the participant stood comfortably with weight evenly distributed on both feet. Blood pressure measurements were recorded from the right arm of seated participants using a digital blood pressure monitor (HEM-705CP, Omron, Tokyo, Japan). Systolic and diastolic blood pressures were recorded three times after a 5 min resting period, with each reading followed by an additional 3 min break. Hypertension was defined as systolic blood pressure exceeding 140 mmHg, diastolic blood pressure exceeding 90 mmHg, or self-reported use of antihypertensive medication based on specific ATC codes (C2, C3, C7, C8, and C9). The definition of diabetes mellitus was based on self-reported physician’s diagnosis, glycated hemoglobin (HbA1c) level of 6.5% or higher, glucose above 11.1 mmol/L, or self-reported use of antidiabetic medication within the past 7 days, specified by the ATC code A10.

Blood samples were collected from the cubital vein while participants were in the supine position between 7 a.m. and 1 p.m. These samples were stored at −80 °C in the Integrated Research Biobank (LiCONiC AG, Mauren, Lichtenstein) of the University Medicine Greifswald in compliance with the appropriate regulations [18]. High-performance liquid chromatography (LC–MS) with spectrophotometric detection (Diamat Analyzer; Bio-Rad, Munich, Germany) was applied to measure HbA1c. Total serum cholesterol, high-density lipoprotein (HDL) cholesterol, low-density lipoprotein (LDL) cholesterol, triglycerides and hsCRP levels were quantified using the Dimension Vista 500 analytical system (Siemens AG, Erlangen, Germany). Serum creatinine levels were determined using an enzymatic method on the same analytical platform. The eGFR was calculated using the four-variable Chronic Kidney Disease Epidemiology Collaboration (CKD-EPI) equation [19]. Fibrinogen concentrations were quantified in citrate plasma samples according to the Clauss method, employing a BCS-XP system (Siemens Healthcare Diagnostics, Eschborn, Germany). WBC concentrations were determined in EDTA whole blood samples using either the Sysmex XT 2000, XE 5000, or SE9000 analyzers (Sysmex, Kobe, Japan) or the Advia 2120i platform (Siemens Healthcare Diagnostics, Erlangen, Germany).

### 2.3. Quantification of Arginine, Asymmetric and Symmetric Dimethylarginine

Liquid chromatography–tandem mass spectrometric (LC–MS/MS) analyses of serum Arg, ADMA and SDMA concentrations were carried out according to previously published protocols established in our laboratory [20]. In brief, 25 μL of serum was diluted in 100 µL of methanol with internal standards added, i.e., stable isotope labeled Arg, SDMA, and ADMA. The samples were then passed through a 0.22 μm hydrophilic membrane for filtration on 96-well plates (Multiscreen HTS™, Millipore, Molsheim, France). After filtration, we evaporated the eluate to dryness and converted the analytes to their butyl ester derivatives with 1 N hydrochloric acid in butanol. After centrifugation, the eluates were dried by heating at 70 °C. Analytes were redissolved in 100 µL of a mixture of methanol and water (25/75, vol/vol) containing 0.1% ammonium formate. Samples were stored at 10 °C in a CTC combi PAL autosampler (CTC Analytics AG, Zwingen, Switzerland) and 10 µL aliquots were injected in the LC–MS/MS system (Varian 1200 MS, Agilent Technologies, Santa Clara, CA, USA) equipped with two Varian ProStar model 210 HPLC pumps. For the separation of analytes, we used a Polaris C18-Ether column (50 × 2.0 mm by Agilent Technolgies, Waldbronn, Germany,) and employed an elution gradient consisting of (A) 0.1% formic acid in water and (B) a mixture of acetonitrile and methanol (50/50, vol/vol) containing 0.1% aqueous formic acid. The elution gradient was as follows: 0:00 min—95/5 (A/B, vol/vol), 0:30 min—95/5, 2:00 min—50/50, 2:01 min—95/5, 4:00 min—95/5. The column temperature was maintained at 30 °C, and the flow rate was set at 0.3 mL/min. For nebulizing and drying, we used nitrogen gas at 250 °C with a flow rate of 90 and 180 L/h, respectively. In the positive electrospray ionization (ESI+) mode, the needle and shield voltage were set at 5000 and 600 V, respectively. To determine the concentrations of guanidino compounds analyzed, we calculated the peak area ratios of the analyte and internal standard using calibration curves based on four levels in triplicates. We also ran plate-wise quality controls (QC) at two levels in duplicates. Furthermore, we conducted a plate-wise second analysis of samples if the coefficient of variation and bias of the QC were above 15%.

### 2.4. Cytokine Measurements

A bead-based assay (Bio-Plex Pro™ Human Inflammation Assay Panel 1, Bio-Rad Laboratories, Hercules, CA, USA) was used to measure a total of 37 inflammatory biomarkers and cytokines in blood plasma. Following the manufacturer’s protocol, 50 μL of the coupled magnetic bead mixture was added to each well in a 96-well plate and washed twice. Subsequently, 50 µL of 1:4 diluted EDTA plasma, serial dilutions of the reconstituted standard, blanks, or controls were added. The plate was then incubated in the dark at room temperature with shaking at 850 rpm overnight. After incubation, the plates were washed three times. Next, a detection antibody mixture was added to the wells and incubated for an additional 30 min at room temperature with shaking at 850 rpm. The plates were washed three times, and streptavidin-PE was added for a 10 min incubation at room temperature with shaking at 850 rpm. The 96-well plates were washed again three times and measurements were performed on a FLEXMAP3D^®^ (Luminex Corp., Austin, TX, USA) platform. To obtain quantitative data, a 5P-logistic regression model was applied to the standard dilution curve data (xPONENT^®^ v4.2 software, Luminex Corp.). Of the 37 inflammatory biomarkers, six with a high number of values below the limit of detection were excluded from the analyses. The remaining biomarkers achieved a median coefficient of variation of 7.7%. As the measurements were conducted in six batches, differences between the plates were corrected using median normalization.

### 2.5. Statistical Analysis

Continuous data are presented as the median (25th, 75th quartile); with nominal data expressed as percentages. The Kruskal–Wallis test was applied for comparison of continuous data and the chi-square test was used to compare nominal data. Linear regression models were calculated to determine the associations between Arg, ADMA or SDMA as well as Arg/ADMA or Arg/SDMA (exposure variables) and inflammatory markers (outcomes, log2-transformed). The beta coefficient (β) and standard error (SE) was calculated for each variable. Models were adjusted for age, sex, WC, smoking status, total cholesterol, diabetes (only whole population) and hypertension. To explore possible nonlinear associations, restricted cubic splines were used. Briefly, in the case of restricted cubic splines, the independent variable is split up in segments defined by the number and location of the so-called knots. In each segment, cubic polynomials are fitted and are chosen so that the spline is continuous/smooth at each knot. For the present analyses, three knots located at the 5th, 50th, and 95th percentiles were defined [21], resulting in one component of the spline function, e.g., arginine’.

If the likelihood ratio test indicated a significant improvement in model fitness, the spline term for Arg, ADMA, SDMA, Arg/ADMA or Arg/SDMA was included in the regression model. For inflammatory markers measured by the cytokine kit, in addition to the *p*-value, Benjamini–Hochberg correction for multiple testing was applied, maintaining the false discovery rate (FDR) at or below 5%. Statistical analyses were performed using SAS 9.4 (SAS Institute Inc., Cary, NC, USA).

## 3. Results

General characteristics for the whole as well as subsample of the SHIP-TREND cohort are given in Table 1. The whole study population was on average 53 years old and thus 4 years older than the subsample, in which an additional 31 inflammatory biomarkers were available. In particular, the frequency of diabetes is lower in the 456 individuals of the subsample as compared to the 3556 participants of the SHIP-TREND cohort investigated in this study (Table 1).

**Figure 1:**
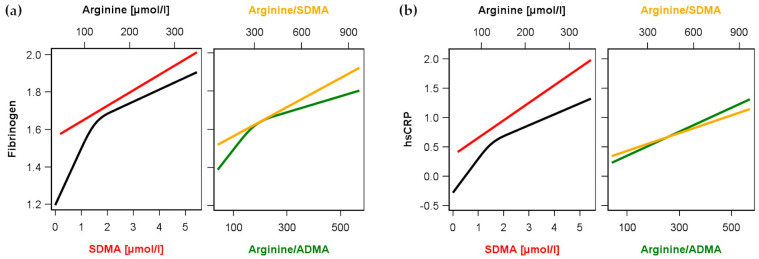
Association between arginine (black) or symmetric dimethylarginine (SDMA, red) as well as the ratios arginine/ADMA (green) and arginine/SDMA (orange) and (**a**) levels of fibrinogen or (**b**) high-sensitivity C-reactive protein (hsCRP) in the whole study population. Linear regression models without or with restricted cubic splines (see methods and Table 2) were adjusted for age, sex, waist circumference, smoking, total cholesterol, diabetes and hypertension.

Multivariable linear regression analyses were used to detect possible associations between Arg, ADMA or SDMA and the investigated established inflammatory markers in the whole population (Table 2 and Figure 1). We observed significant positive associations of Arg and SDMA but not of ADMA with hsCRP as well as fibrinogen in the whole study population (n = 3556). With respect to the ratios, Arg/ADMA as well as Arg/SDMA were both positive linked to hsCRP and fibrinogen. We found that Arg, ADMA, or SDMA as well as Arg/ADMA and Arg/SDMA were not significantly associated with WBC.

**Figure 2:**
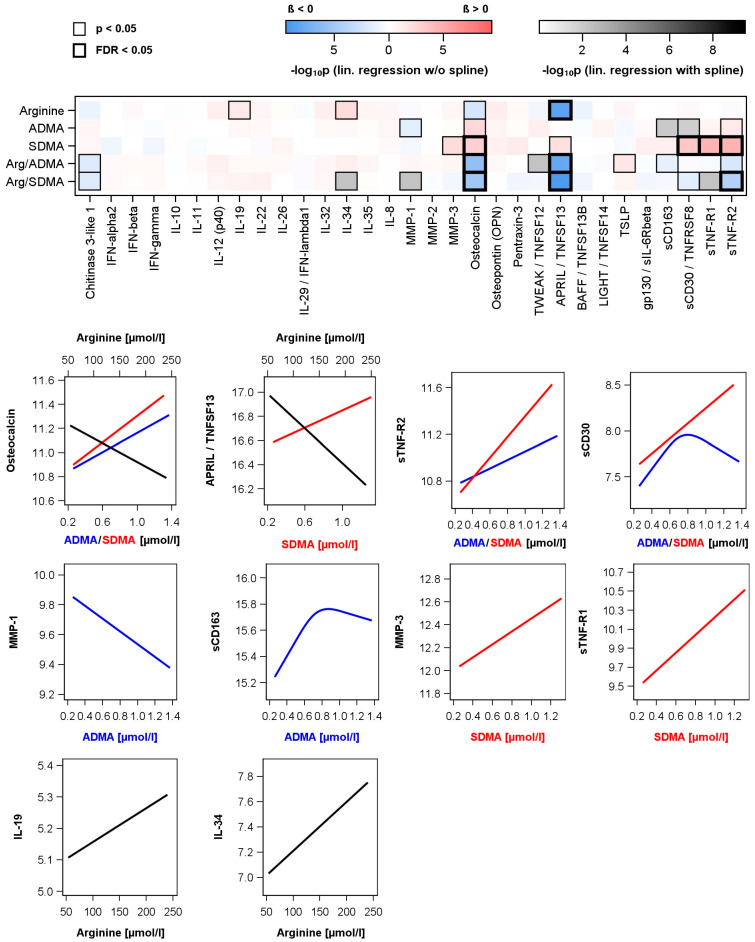
Associations between arginine, asymmetric (ADMA) or symmetric (SDMA) dimethylarginine as well as ratios and levels of measured cytokines and inflammatory biomarkers in the subpopulation. Upper part: Color-coded *p*-value from linear regression models without or with restricted cubic splines (see methods and Table S1) adjusted for age, sex, waist circumference, smoking, total cholesterol and hypertension. Significant associations with *p*-values or corrected *p*-values (controlling the false discovery rate (FDR)) < 0.05) are marked with a black box. Lower part: Regression lines for significant associations (black—arginine; red—SDMA; blue—ADMA). Regression lines for significant ratios are given in Figure S1.

In the subsample (n = 456) used for the investigation of relations between Arg, ADMA or SDMA and cytokines or markers of inflammation, various associations were detected (Figure 2 and Table S1). Arg was positively related to interleukin (IL)-19 and IL-34 as well as inversely related to osteocalcin and a proliferation-inducing ligand/tumor necrosis factor (TNF) superfamily member 13 (TNFSF13), also known as a proliferation-inducing ligand (APRIL). Regarding SDMA only positive associations were observed. Again, osteocalcin and APRIL/TNFSF13 were found with opposite direction compared to Arg. Additional, SDMA concentrations were positively linked to matrix metalloproteinase (MMP)-3, TNF receptor superfamily member 8 (TNFRSF8) also known as soluble cluster of differentiation (sCD) 30, soluble TNF receptor (sTNF-R)1 and sTNF-R2. ADMA showed similar but weaker linear associations to osteocalcin and sTNF-R2 as well as an inverse relation to MMP-1. Furthermore, ADMA showed inverted reverse J-shaped relations to sCD30 and sCD163. With respect to the Arg/ADMA or Arg/SDMA ratio, we observed significant associations with the same markers whereby the association direction is mainly triggered by arginine.

## 4. Discussion

In the present study, we identified Arg and SDMA but not ADMA to be positively associated with common but unspecific markers of inflammation in the general population of the SHIP-TREND study. Furthermore, in a subsample including a broad set of cytokines Arg, SDMA and ADMA were related to different pro-inflammatory cytokines. Coming from an ICU perspective, the results are difficult to interpret and our aim was challenging. The incidence of life-threatening acute disease treated in ICU in a population-based cohort such as SHIP-TREND is, per definition, zero percent. Therefore, the aim was not to answer the question whether Arg, SDMA and ADMA are associated with acute life-threating disease. This has been previously shown for an ICU cohort of sepsis patients [3]. Thus, it is more important and interesting to measure NO-related markers in this large population-based cohort for mainly two other reasons: (1) Is NO metabolism associated with common inflammatory markers often used in an outpatient setting and screening? (2) Is a cytokine-driven immune response in a non-critically ill cohort related to NO metabolism?

Answering both questions is a first step not only to define normal ranges for Arg, ADMA and SDMA to better interpret those levels in an ICU setting but also fundamental to strengthen our previous observation that Arg, ADMA and SDMA may play a role as alternative markers for acute inflammatory disease and septic shock. To our knowledge, this is the first study and approach in which lately discovered ICU biomarkers related to sepsis and shock are measured in a non-critically ill cohort and without sepsis.

Arg is a substrate of NOS while ADMA is an endogenous inhibitor of NOS. Three isoforms of NOS exists, neuronal NOS (nNOS) and endothelial (eNOS) are expressed in the vascular wall constitutively while the inducible NOS (iNOS) is expressed under inflammatory conditions [22]. Even though Arg is essential to maintain vascular homeostasis of vasodilation and vasoconstriction, under inflammatory stimulation vast NO production by iNOS may be detrimental [23]. Of note, arginine supplementation of patients after myocardial infarction was found to be a risk for mortality and morbidity in one study, whereas no effect at least on mortality rate was observed in hypertensive patients [24,25]. The association of Arg and hsCRP might therefore be indirect, identifying individuals with subclinical inflammation. This notion is in line with previous findings in atherosclerotic patients showing a positive correlation of arginine and hsCRP [26]. This might be explained at least in part by an alteration of Arg metabolism in obese individuals with mild inflammation [27]. In the subgroup of 456 SHIP-TREND participants, Arg was positively related to IL-19 and IL-34 but inversely related to APRIL/TNFSF13. The protein APRIL, which belongs to the TNF superfamily, is released into the bloodstream in response to inflammatory signals such as IFN-gamma or IFN-beta. The release is triggered by molecules associated with pathogens, i.e., damage-associated molecular patterns (PAMPs) or damage-associated molecular patterns (DAMPs), which are recognized by specific receptors called pattern recognition receptors (PRRs) including various Toll-like receptors (TLRs) [28]. In the pathophysiology of cardiovascular disease, APRIL is linked to the development of atherosclerosis [29]. Mechanistically, APRIL is atheroprotective by binding to heparan sulfate chains of heparan-sulfate proteoglycan 2. This interaction reduces the accumulation of LDL cholesterol, invasion of macrophages and thereby limits the formation of necrotic cores. Recent studies have also associated APRIL with insulin resistance, lipolysis, brown adipose tissue dependent thermogenesis and nonalcoholic fatty liver disease, suggesting a broader involvement in energy metabolism [28,30].

The link between SDMA and inflammation seems to be independent of NOS. SDMA was previously shown to induce TNFα expression in monocytes by nuclear factor (NF)-κB activation [31]. We found that SDMA also associates with soluble TNF receptor (superfamily) concentrations, i.e., sTNF-R1, sTNF-R2, and sCD30/TNFRSF8 in a subsample of the population-based SHIP-TREND study, indicating that this interaction is not only evident at higher SDMA concentrations seen in patients with chronic kidney disease but also could be relevant within the normal range of SDMA concentrations, i.e., 0.27 and 0.63 µmol/L, 2.5th and 97.5th percentile, respectively, in women and 0.30 and 0.67 µmol/L, 2.5th and 97.5th percentile, respectively, in men [11]. We previously identified a strong inverse association of sTNF-R2 with the HDL subclasses Apo-A1 and Apo-A2 in the SHIP-TREND cohort [30]. SDMA binds to Apo-A1 and Apo-A2 and thereby modifies the properties of HDL particles. In children and adults with chronic kidney disease (CKD), a population with high cardiovascular risk, Speer et al. have demonstrated that HDL from these patients, i.e., HDL(CKD), contrast to HDL from healthy donors promoted endothelial superoxide production, substantially reduced NO bioavailability, and subsequently increased arterial blood pressure [32]. Increased circulating concentrations of SDMA in these patients transformed HDL into HDL(CKD). This HDL(CKD) promoted endothelial dysfunction. Mechanistically, SDMA bound to HDL reduced endothelial NO availability via Toll-like receptor-2 (TLR-2), leading to impaired endothelial repair, increased proinflammatory activation, and hypertension. TLR2 is another well-known trigger of immune responses in the arterial wall, leading to the induction of the inflammasome including the release pro-inflammatory cytokines, such as IL-1β [33]. Furthermore, studies have shown that TLR-2 plays a significant role in driving inflammation in human atherosclerosis through a signaling adaptor called MyD88 [34]. Collectively, this evidence strongly suggests that TLR-2 is one of the most proatherogenic TLRs.

SDMA was previously found to be associated with vertebral fractures and serum osteocalcin in type 2 diabetic patients [35]. This suggests a potential role of SDMA in bone turnover. However, to date, there is no mechanistic or causative evidence for this notion. SDMA activates NF-κB (see above) and associates with several receptors of the TNF (superfamily). The TNF (superfamily) ligand interaction with the TNF (superfamily) receptor leads to a downstream intracellular signaling which is amplified by the recruitment of a number of adaptors including TNF receptor associated factor (TRAF). The receptor activator of NF-κB (RANK) to RANK ligand (RANKL) signaling pathway is mediated by TRAF6 recruitment, comprising the master regulator of the osteoclast-specific transcriptional activation [36]. Interestingly enough, recent research has identified the Jumonji C domain-containing protein (JMJD)6 to demethylate the methylation of arginine residues of the TRAF6 protein resulting in maximal activation of NF-κB and release of methylated arginine residues [37].

As compared with SDMA, ADMA showed similar but weaker associations to osteocalcin and sTNF-R2. Furthermore, an inverse relation of ADMA to MMP-1 was observed. This may suggest, that beyond a relation to the immune system, ADMA has an even stronger link to the vascular system in particular to vascular remodeling seen in intimal hyperplasia and atherosclerosis. There is evidence from several cohorts that the risk for cardiovascular events, i.e., from myocardial infarction and stroke is predicted by ADMA independently of hypertension, obesity and hypertriglyceridemia [38]. In line with this interpretation, we and others recently identified higher levels of ADMA to be associated with the presence of subclinical atherosclerosis as defined by coronary artery calcification (CAC) and carotid intimal media thickness (cIMT) in other community-based studies [39,40,41]. However, also Arg and SDMA have been linked to subclinical atherosclerosis, i.e., cIMT [42]. Taking together these observations from the SHIP-TREND cohort and other studies, this suggests that SDMA and ADMA are not only markers of atherosclerosis but are also mediators of cardiovascular impairment.

Due to the cross-sectional study design and single-time guanidino compound and cytokine measurement, we can only speculate whether our observations are based on an asymptomatic acute infection or chronic low-grade inflammation. Also no direct mechanistic evidence can be drawn from our observations and all associations seen can only foster future basic science research to provide causality.

## 5. Conclusions

Our findings contribute new insights into the involvement of Arg, ADMA, and SDMA in subclinical inflammation in the general population. In particular, SDMA is associated with subclinical inflammation in the general population. For Arg derivatives, mechanisms other than NO might be more relevant as triggers of subclinical inflammation and deserve further investigation. It needs to be investigated whether SDMA and Arg are also predictive for unfavorable outcomes in patients with infectious disease. In this context, a longitudinal prospective study design including patients at risk may focus on this particular aspect, e.g., in emergency departments.

## Figures and Tables

**Table 1:** General characteristics of the used study populations.

	Whole Population n = 3556	Subpopulation n = 456
Men, %	50.7	46.7
Age, years	53 (40; 64)	49 (40; 60)
Smoking, %		
Never smoker	36.0	42.3
Ex-smoker	37.2	34.2
Current smoker	26.8	23.5
Diabetes, %	12.2	3.3
Hypertension, %	47.8	37.3
Waist circumference, cm	91 (81; 101)	88 (80; 97)
BMI, kg/m^2^	27.6 (24.6; 31.0)	26.9 (24.2; 30.2)
Total cholesterol, mmol/L	5.4 (4.6; 6.2)	5.5 (4.8; 6.2)
HDL cholesterol, mmol/L	1.39 (1.16; 1.66)	1.42 (1.20; 1.67)
LDL cholesterol, mmol/L	3.31 (2.67; 3.96)	3.40 (2.78; 3.99)
Triglycerides, mmol/L	1.40 (0.96; 2.01)	1.25 (0.88; 1.72)
Creatinine, µmol/L	70 (61; 80)	70 (61; 78)
eGFR, mL/min/1.73 m^2^	97 (85; 108)	98 (89; 108)
WBC, Gpt/L	5.8 (4.9; 7.0)	5.5 (4.7; 6.8)
Fibrinogen, g/L	3.0 (2.5; 3.5)	3.0 (2.4; 3.5)
hsCRP, mg/L	1.29 (0.67; 2.90)	1.18 (0.56; 3.22)
Arginine, µmol/L	118 (105; 134)	121 (107; 137)
ADMA, µmol/L	0.70 (0.60; 0.81)	0.70 (0.60; 0.81)
SDMA, µmol/L	0.50 (0.42; 0.60)	0.52 (0.44; 0.62)

Abbreviations: ADMA, asymmetric dimethylarginine; BMI, body mass index; eGFR, estimated glomerular filtration rate; HDL, high-density lipoprotein; hsCRP, high-sensitivity C-reactive protein; LDL, low-density lipoprotein; SDMA, symmetric dimethylarginine; WBC, white blood count.

**Table 2:** Associations between arginine or arginine derivate and markers of inflammation in the whole study population.

	White Blood Count			Fibrinogen			High-Sensitivity C-Reactive Protein		
	β	SE	*p*	β	SE	*p*	β	SE	*p*
Arginine Arginine’	2.44 × 10^−4^	2.44 × 10^−4^	0.32	5.23 × 10^−3^	4.75 × 10^−4^	<0.01	0.010	0.002	<0.01
	-	-	-	−5.64 × 10^−7^	9.59 × 10^−8^	<0.01	−9.29 × 10^−7^	4.32 × 10^−7^	0.03
ADMA	−0.010	0.028	0.71	0.008	0.022	0.71	0.060	0.098	0.54
SDMA	−0.018	0.038	0.64	0.083	0.031	0.01	0.298	0.137	0.03
Arginine/ADMA	1.93 × 10^−4^	1.21 × 10^−4^	0.11	1.79 × 10^−3^	2.54 × 10^−4^	<0.01	2.04 × 10^−3^	4.30 × 10^−4^	<0.01
Arginine/ADMA’	-	-	-	−4.75 × 10^−8^	1.31 × 10^−8^	<0.01	-	-	-
Arginine/SDMA	1.29 × 10^−4^	7.81 × 10^−5^	0.10	4.44 × 10^−4^	6.28 × 10^−5^	<0.01	8.83 × 10^−4^	2.78 × 10^−4^	<0.01

Abbreviations: ADMA, asymmetric dimethylarginine; β, beta coefficient; SE, standard error; SDMA, symmetric dimethylarginine; Stderr, standard error. Arginine’ and arginine/ADMA’ are spline components (more details see method Section 2.5). Linear regression analyses were adjusted for age, sex, waist circumference, smoking, total cholesterol, diabetes and hypertension.

## Data Availability

The data presented in this study are available on request by a qualified investigator for 3 years after the date of publication from the corresponding author.

## Supplementary Materials

The following supporting information can be downloaded at: https://www.mdpi.com/article/10.3390/biom13111612/s1, Figure S1: Flow diagram for selection of study population; Table S1: Associations between arginine or arginine derivate and measured cytokines in the subpopulation.

## References

[1] Böger R.H. (2006). Live and let die: Asymmetric dimethylarginine and septic shock. Crit. Care.

[2] Bogdan C. (2015). Nitric oxide synthase in innate and adaptive immunity: An update. Trends Immunol..

[3] Winkler M.S., Nierhaus A., Rösler G., Lezius S., Harlandt O., Schwedhelm E., Böger R.H., Kluge S. (2018). Symmetrical (SDMA) and asymmetrical dimethylarginine (ADMA) in sepsis: High plasma levels as combined risk markers for sepsis survival. Crit. Care.

[4] Singer M., Deutschman C.S., Seymour C.W., Shankar-Hari M., Annane D., Bauer M., Bellomo R., Bernard G.R., Chiche J.D., Coopersmith C.M. (2016). The Third International Consensus Definitions for Sepsis and Septic Shock (Sepsis-3). JAMA.

[5] Seymour C.W., Liu V.X., Iwashyna T.J., Brunkhorst F.M., Rea T.D., Scherag A., Rubenfeld G., Kahn J.M., Shankar-Hari M., Singer M. (2016). Assessment of Clinical Criteria for Sepsis: For the Third International Consensus Definitions for Sepsis and Septic Shock (Sepsis-3). JAMA.

[6] Denegri A., Boriani G. (2021). High Sensitivity C-reactive Protein (hsCRP) and its Implications in Cardiovascular Outcomes. Curr. Pharm. Des..

[7] Bauer M., Gerlach H., Vogelmann T., Preissing F., Stiefel J., Adam D. (2020). Mortality in sepsis and septic shock in Europe, North America and Australia between 2009 and 2019- results from a systematic review and meta-analysis. Crit. Care.

[8] O’Dwyer M.J., Dempsey F., Crowley V., Kelleher D.P., McManus R., Ryan T. (2006). Septic shock is correlated with asymmetrical dimethyl arginine levels, which may be influenced by a polymorphism in the dimethylarginine dimethylaminohydrolase II gene: A prospective observational study. Crit. Care.

[9] Mortensen K.M., Itenov T.S., Haase N., Müller R.B., Ostrowski S.R., Johansson P.I., Olsen N.V., Perner A., Søe-Jensen P., Bestle M.H. (2016). High levels of methylarginines were associated with increased mortality in patients with severe sepsis. Shock.

[10] van Wijk X.M.R., Yun C., Lynch K.L. (2021). Evaluation of biomarkers in sepsis: High dimethylarginine (ADMA and SDMA) concentrations are associated with mortality. J. Appl. Lab. Med..

[11] Atzler D., Schwedhelm E., Nauck M., Ittermann T., Böger R.H., Friedrich N. (2014). Serum reference intervals of homoarginine, ADMA, and SDMA in the study of health in Pomerania. Clin. Chem. Lab. Med..

[12] Moritz E., Jedlitschky G., Negnal J., Tzvetkov M.V., Daum G., Dörr M., Felix S.B., Völzke H., Nauck M., Schwedhelm E. (2021). Increased Sphingosine-1-Phosphate Serum Concentrations in Subjects with Periodontitis: A Matter of Inflammation. J. Inflamm. Res..

[13] Rotheudt L., Moritz E., Markus M.R.P., Albrecht D., Völzke H., Friedrich N., Schwedhelm E., Daum G., Schminke U., Felix S.B. (2022). Sphingosine-1-phosphate and vascular disease in the general population. Atherosclerosis.

[14] Ponce-de-Leon M., Hannemann A., Linseisen J., Nauck M., Lerch M.M., Bülow R., Völzke H., Friedrich N., Kassubek J., Müller H.P. (2022). Links between ectopic and abdominal fat and systemic inflammation: New insights from the SHIP-Trend study. Dig. Liver Dis..

[15] Völzke H., Alte D., Schmidt C.O., Radke D., Lorbeer R., Friedrich N., Aumann N., Lau K., Piontek M., Born G. (2011). Cohort Profile: The Study of Health in Pomerania. Int. J. Epidemiol..

[16] Völzke H., Schössow J., Schmidt C.O., Jürgens C., Richter A., Werner A., Werner N., Radke D., Teumer A., Ittermann T. (2022). Cohort Profile Update: The Study of Health in Pomerania (SHIP). Int. J. Epidemiol..

[17] Völzke H., Ittermann T., Schmidt C.O., Baumeister S.E., Schipf S., Alte D., Biffar R., John U., Hoffmann W. (2015). Prevalence trends in lifestyle—Related risk factors. Dtsch. Ärzteblatt Int..

[18] Winter T., Friedrich N., Lamp S., Schäfer C., Schattschneider M., Bollmann S., Brümmer D., Riemann K., Petersmann A., Nauck M. (2020). The Integrated Research Biobank of the University Medicine Greifswald. Open J. Bioresour..

[19] Kuster N., Cristol J.-P., Cavalier E., Bargnoux A.-S., Halimi J.-M., Froissart M., Piéroni L., Delanaye P. (2014). Enzymatic creatinine assays allow estimation of glomerular filtration rate in stages 1 and 2 chronic kidney disease using CKD-EPI equation. Clin. Chim. Acta.

[20] Schwedhelm E., Maas R., Tan-Andresen J., Schulze F., Riederer U., Böger R.H. (2007). High-throughput liquid chromatographic-tandem mass spectrometric determination of arginine and dimethylated arginine derivatives in human and mouse plasma. J. Chromatogr. B.

[21] Stone C.J., Koo C.Y. (1985). Additive splines in statistics. Proc. Stat. Comp. Sect. Am. Stat. Assoc..

[22] Pautz A., Li H., Kleinert H. (2021). Regulation of NOS expression in vascular diseases. Front. Biosci..

[23] Feihl F., Waeber B., Liaudet L. (2001). Is nitric oxide overproduction the target of choice for the management of septic shock?. Pharmacol. Ther..

[24] Schulman S.P., Becker L.C., Kass D.A., Champion H.C., Terrin M.L., Forman S., Ernst K.V., Kelemen M.D., Townsend S.N., Capriotti A. (2006). L-arginine therapy in acute myocardial infarction: The Vascular Interaction with Age in Myocardial Infarction (VINTAGE MI) randomized clinical trial. JAMA.

[25] Menzel D., Haller H., Wilhelm M., Robenek H. (2018). L-Arginine and B vitamins improve endothelial function in subjects with mild to moderate blood pressure elevation. Eur. J. Nutr..

[26] Zhu Q., Wu Y., Mai J., Guo G., Meng J., Fang X., Chen X., Liu C., Zhong S. (2022). Comprehensive Metabolic Profiling of Inflammation Indicated Key Roles of Glycerophospholipid and Arginine Metabolism in Coronary Artery Disease. Front. Immunol..

[27] Wierzchowska-McNew R.A., Engelen M.P.K.J., Thaden J.J., Ten Have G.A.M., Deutz N.E.P. (2022). Obesity- and sex-related metabolism of arginine and nitric oxide in adults. Am. J. Clin. Nutr..

[28] Baert L., Ahmed M.C., Manfroi B., Huard B. (2021). The number 13 of the family: A proliferation inducing ligand. Curr. Opin. Immunol..

[29] Tsiantoulas D., Eslami M., Obermayer G., Clement M., Smeets D., Mayer F.J., Kiss M.G., Enders L., Weißer J., Göderle L. (2021). APRIL limits atherosclerosis by binding to heparan sulfate proteoglycans. Nature.

[30] Pontzen D.L., Bahls M., Albrecht D., Felix S.B., Dörr M., Ittermann T., Nauck M., Friedrich N. (2023). Low-grade inflammation is associated with a heterogeneous lipoprotein subclass profile in an apparently healthy population sample. Lipids Health Dis..

[31] Schepers E., Barreto D.V., Liabeuf S., Glorieux G., Eloot S., Barreto F.C., Massy Z., Vanholder R. (2011). European Uremic Toxin Work Group (EUTox). Symmetric dimethylarginine as a proinflammatory agent in chronic kidney disease. Clin. J. Am. Soc. Nephrol..

[32] Speer T., Rohrer L., Blyszczuk P., Shroff R., Kuschnerus K., Kränkel N., Kania G., Zewinger S., Akhmedov A., Shi Y. (2013). Abnormal high-density lipoprotein induces endothelial dysfunction via activation of Toll-like receptor-2. Immunity.

[33] Dinarello C.A. (2011). Interleukin-1 in the pathogenesis and treatment of inflammatory diseases. Blood.

[34] Monaco C., Gregan S.M., Navin T.J., Foxwell B.M., Davies A.H., Feldmann M. (2009). Toll-like receptor-2 mediates inflammation and matrix degradation in human atherosclerosis. Circulation.

[35] Kanazawa I., Yano S., Yamaguchi T., Notsu Y., Nabika T., Sugimoto T. (2010). Relationships between dimethylarginine and the presence of vertebral fractures in type 2 diabetes mellitus. Clin. Endocrinol..

[36] Kobayashi N., Kadono Y., Naito A., Matsumoto K., Yamamoto T., Tanaka S., Inoue J. (2001). Segregation of TRAF6-mediated signaling pathways clarifies its role in osteoclastogenesis. EMBO J..

[37] Tikhanovich I., Kuravi S., Artigues A., Villar M.T., Dorko K., Nawabi A., Roberts B., Weinman S.A. (2015). Dynamic Arginine Methylation of Tumor Necrosis Factor (TNF) Receptor-associated Factor 6 Regulates Toll-like Receptor Signaling. J. Biol. Chem..

[38] Dowsett L., Higgins E., Alanazi S., Alshuwayer N.A., Leiper F.C., Leiper J. (2020). ADMA: A Key Player in the Relationship between Vascular Dysfunction and Inflammation in Atherosclerosis. J. Clin. Med..

[39] Iribarren C.H.G., Sydow K., Wang B.Y., Sidney S., Cooke J.P. (2007). Asymmetric dimethyl-arginine and coronary artery calcification in young adults entering middle age: The CARDIA Study. Eur. J. Prev. Cardiol..

[40] Chirinos J.A., David R., Bralley J.A., Zea-Díaz H., Muñoz-Atahualpa E., Corrales-Medina F., Cuba-Bustinza C., Chirinos-Pacheco J., Medina-Lezama J. (2008). Endogenous nitric oxide synthase inhibitors, arterial hemodynamics, and subclinical vascular disease: The PREVENCION Study. Hypertension.

[41] Maas R., Xanthakis V., Polak J.F., Schwedhelm E., Sullivan L.M., Benndorf R., Schulze F., Vasan R.S., Wolf P.A., Böger R.H. (2009). Association of the endogenous nitric oxide synthase inhibitor ADMA with carotid artery intimal media thickness in the Framingham Heart Study offspring cohort. Stroke.

[42] Bahls M., Friedrich N., Atzler D., Felix S.B., Nauck M.A., Böger R.H., Völzke H., Schwedhelm E., Dörr M. (2015). L-Arginine and SDMA Serum Concentrations Are Associated with Subclinical Atherosclerosis in the Study of Health in Pomerania (SHIP). PLoS ONE.

