# Supplementary material for: Association of Asymmetric and Symmetric Dimethylarginine with Inflammation in the Population-Based Study of Health in Pomerania
Source: Biomolecules. 2023 Nov 4;13(11):1612. doi: 10.3390/biom13111612 (PMC10669713; doi:10.3390/biom13111612)
Supplement: Supplementary file 1 [file biomolecules-13-01612-s001.zip › biomolecules-2595560-supplementary.pdf]

SUPPLEMENT

Table S1. Associations between arginine or arginine derivate as well as ratios and measured cytokines in the sub population.

	Arginine		ADMA		ADMA’		SDMA		Arginine/ADMA		Arginine/ADMA’		Arginine/SDMA		Arginine/SDMA’	
(Log2-transformed)	Beta (stderr)	p	Beta (stderr)	p	Beta (stderr)	p	Beta (stderr)	p	Beta (stderr)	p	Beta (stderr)	p	Beta (stderr)	p	Beta (stderr)	p
**APRIL / TNFSF13**	**-3.99E^{-03} (6.60E^{-04})**	**<.01**	0.056 (0.115)	0.63			**0.356 (0.141)**	**0.01**	**-2.05E^{-03} (3.50E^{-04})**	**<.01**			**-1.54E^{-03} (2.44E^{-04})**	**<.01**		
**BAFF / TNFSF13B**	-1.26E^{-03} (7.30E^{-04})	0.08	-0.084 (0.123)	0.50			5.63E^{-03} (0.151)	0.97	-1.72E^{-04} (3.88E^{-04})	0.66			-4.38E^{-04} (2.71E^{-04})	0.11		
**Chitinase 3-like 1**	-1.20E^{-03} (6.91E^{-04})	0.08	0.123 (0.116)	0.29			0.160 (0.143)	0.26	**-9.09E^{-04} (3.64E^{-04})**	**0.01**			**-6.46E^{-04} (2.56E^{-04})**	**0.01**		
**gp130 / sIL-6Rbeta**	1.15E^{-04} (9.07E^{-04})	0.90	0.100 (0.152)	0.51			0.303 (0.187)	0.11	-3.06E^{-04} (4.80E^{-04})	0.52			-4.54E^{-04} (3.36E^{-04})	0.18		
**IFN-alpha2**	6.18E^{-05} (7.10E^{-04})	0.93	-0.020 (0.119)	0.86			-0.212 (0.146)	0.15	2.58E^{-04} (3.75E^{-04})	0.49			1.91E^{-04} (2.63E^{-04})	0.47		
**IFN-beta**	1.63E^{-03} (1.66E^{-03})	0.33	-0.106 (0.284)	0.71			0.031 (0.361)	0.93	7.98E^{-04} (9.47E^{-04})	0.40			3.47E^{-04} (6.27E^{-04})	0.58		
**IFN-gamma**	1.67E^{-04} (8.80E^{-04})	0.85	-0.084 (0.148)	0.57			-0.248 (0.182)	0.17	4.20E^{-04} (4.65E^{-04})	0.37			2.85E^{-04} (3.27E^{-04})	0.38		
**IL-10**	-1.64E^{-04} (4.06E^{-04})	0.69	0.028 (0.068)	0.68			0.032 (0.084)	0.70	-6.22E^{-05} (2.15E^{-04})	0.77			-3.05E^{-05} (1.51E^{-04})	0.84		
**IL-11**	8.23E^{-04} (1.99E^{-03})	0.68	-0.073 (0.328)	0.82			-0.241 (0.404)	0.55	8.09E^{-04} (1.06E^{-03})	0.45			2.78E^{-04} (7.54E^{-04})	0.71		
**IL-12 (p40)**	1.69E^{-03} (1.05E^{-03})	0.11	0.020 (0.177)	0.91			-0.078 (0.217)	0.72	8.36E^{-04} (5.56E^{-04})	0.13			4.37E^{-04} (3.90E^{-04})	0.26		
**IL-19**	**1.08E^{-03} (5.26E^{-04})**	**0.04**	0.099 (0.089)	0.27			0.014 (0.109)	0.90	2.45E^{-04} (2.80E^{-04})	0.38			2.68E^{-04} (1.96E^{-04})	0.17		
**IL-22**	9.79E^{-04} (9.03E^{-04})	0.28	0.100 (0.152)	0.51			-0.155 (0.187)	0.41	3.59E^{-04} (4.78E^{-04})	0.45			5.15E^{-04} (3.35E^{-04})	0.12		
**IL-26**	2.76E^{-04} (5.04E^{-04})	0.58	0.024 (0.085)	0.78			0.119 (0.104)	0.25	1.80E^{-04} (2.66E^{-04})	0.50			-2.41E^{-05} (1.87E^{-04})	0.90		
**IL-29 / IFN-lambda1**	-7.01E^{-04} (1.06E^{-03})	0.51	-3.88E^{-03} (0.177)	0.98			0.021 (0.218)	0.92	-4.20E^{-04} (5.58E^{-04})	0.45			-3.66E^{-04} (3.91E^{-04})	0.35		
**IL-32**	3.08E^{-03} (1.93E^{-03})	0.11	0.246 (0.330)	0.46			-7.54E^{-03} (0.411)	0.99	1.06E^{-03} (1.02E^{-03})	0.30			8.18E^{-04} (7.33E^{-04})	0.27		
**IL-34**	**3.88E^{-03} (1.43E^{-03})**	**<.01**	0.228 (0.242)	0.35			-8.19E^{-03} (0.300)	0.98	1.33E^{-03} (7.59E^{-04})	0.08			**3.94E^{-03} (1.36E^{-03})**	**<.01**	**-6.55E^{-08} (2.65E^{-08})**	**0.01**
**IL-35**	9.73E^{-04} (8.54E^{-04})	0.26	0.072 (0.144)	0.62			0.052 (0.177)	0.77	4.87E^{-04} (4.52E^{-04})	0.28			1.72E^{-04} (3.17E^{-04})	0.59		
**IL-8**	8.63E^{-04} (8.18E^{-04})	0.29	0.153 (0.137)	0.27			0.122 (0.169)	0.47	1.08E^{-04} (4.33E^{-04})	0.80			9.52E^{-05} (3.04E^{-04})	0.75		
**LIGHT / TNFSF14**	7.32E^{-04} (1.96E^{-03})	0.71	0.427 (0.330)	0.20			0.101 (0.407)	0.80	-9.60E^{-04} (1.06E^{-03})	0.36			9.44E^{-05} (7.32E^{-04})	0.90		
**MMP-1**	**-1.01E^{-03} (1.13E^{-03})**	**0.37**	**-0.428 (0.192)**	**0.03**			-0.305 (0.243)	0.21	6.64E^{-04} (6.14E^{-04})	0.28			**2.18E^{-03} (1.07E^{-03})**	**0.04**	**-4.72E^{-08} (2.09E^{-08})**	**0.02**
**MMP-2**	3.97E^{-04} (8.39E^{-04})	0.64	-0.033 (0.141)	0.81			0.086 (0.173)	0.62	3.38E^{-04} (4.44E^{-04})	0.45			-1.84E^{-04} (3.11E^{-04})	0.56		
**MMP-3**	1.18E^{-03} (9.98E^{-04})	0.24	0.096 (0.168)	0.57			**0.566 (0.205)**	**<.01**	6.30E^{-04} (5.28E^{-04})	0.23			-4.82E^{-04} (3.70E^{-04})	0.19		
**Osteocalcin**	**-2.34E^{-03} (8.27E^{-04})**	**<.01**	**0.400 (0.139)**	**<.01**			**0.548 (0.171)**	**<.01**	**-2.10E^{-03} (4.30E^{-04})**	**<.01**			**-1.38E^{-03} (3.03E^{-04})**	**<.01**		
**Osteopontin (OPN)**	1.90E^{-03} (1.03E^{-03})	0.06	0.226 (0.173)	0.19			0.318 (0.213)	0.14	3.86E^{-04} (5.46E^{-04})	0.48			1.07E^{-04} (3.83E^{-04})	0.78		
**Pentraxin-3**	9.61E^{-04} (1.24E^{-03})	0.44	0.117 (0.208)	0.57			0.494 (0.254)	0.05	1.80E^{-05} (6.54E^{-04})	0.98			-6.94E^{-04} (4.58E^{-04})	0.13		
**sCD163**	3.16E^{-04} (1.03E^{-03})	0.76	**1.15 (0.406)**	**<.01**	**-3.90 (1.88)**	**0.04**	0.358 (0.211)	0.09	**-1.09E^{-03} (5.41E^{-04})**	**0.04**			-4.22E^{-04} (3.80E^{-04})	0.27		
**sCD30 / TNFRSF8**	1.12E^{-03} (1.13E^{-03})	0.32	**1.32 (0.448)**	**<.01**	**-5.53 (2.08)**	**<.01**	**0.825 (0.231)**	**<.01**	-5.23E^{-04} (5.98E^{-04})	0.38			**-9.28E^{-04} (4.18E^{-04})**	**0.03**		
**sTNF-R1**	7.44E^{-05} (1.09E^{-03})	0.95	0.243 (0.183)	0.19			**0.933 (0.221)**	**<.01**	-5.44E^{-04} (5.76E^{-04})	0.35			**-3.23E^{-03} (1.01E^{-03})**	**<.01**	**4.58E^{-08} (1.99E^{-08})**	**0.02**
**sTNF-R2**	-8.99E^{-04} (1.03E^{-03})	0.38	**0.362 (0.172)**	**0.04**			**0.882 (0.209)**	**<.01**	**-1.49E^{-03} (5.41E^{-04})**	**<.01**			**-1.51E^{-03} (3.76E^{-04})**	**<.01**		
**TSLP**	9.85E^{-04} (9.48E^{-04})	0.30	-0.175 (0.159)	0.27			-0.196 (0.196)	0.32	**1.06E^{-03} (5.00E^{-04})**	**0.03**			4.37E^{-04} (3.52E^{-04})	0.22		
**TWEAK / TNFSF12**	-8.18E^{-04} (6.25E^{-04})	0.19	0.177 (0.105)	0.09			0.042 (0.129)	0.75	**-2.66E^{-03} (8.40E^{-04})**	**<.01**	**1.05E-07 (4.02E^{-08})**	**<.01**	-3.11E^{-04} (2.32E^{-04})	0.18		

ADMA, asymmetric dimethylarginine; SDMA, symmetric dimethylarginine; Stderr, standard error. ADMA’, Arginine/ADMA’ and Arginine/SDMA’ represent spline components (more details see method section). Linear regression were adjusted for age, sex, waist circumference, smoking, total cholesterol and hypertension.

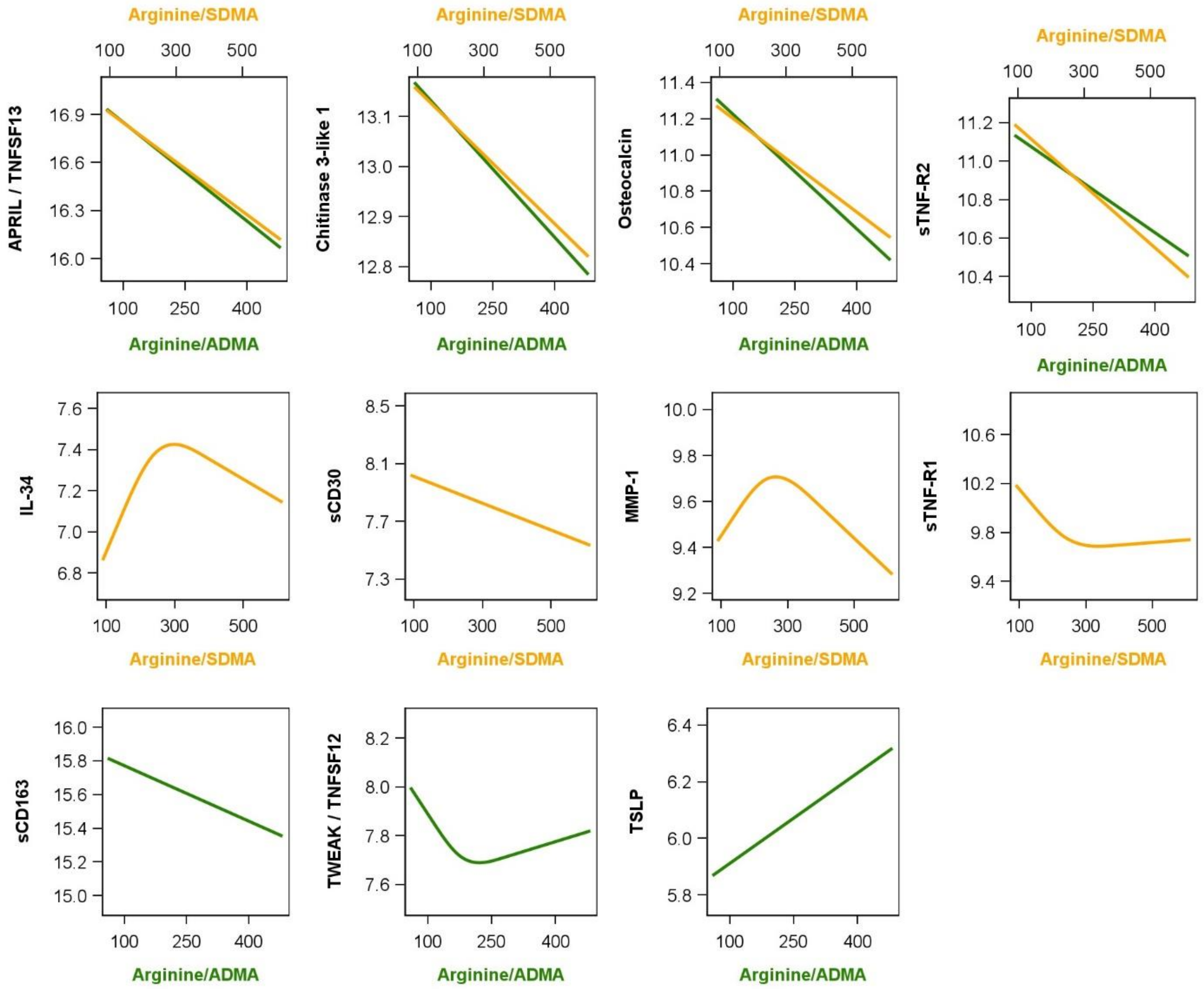

Figure S1. Significant associations between arginine/asymmetric (ADMA - green) or arginine/symmetric (SDMA - orange) dimethylarginine ratio and levels of measured cytokines and inflammatory biomarkers in the subpopulation. Shown are regression lines based on linear regression adjusted for age, sex, waist circumference, smoking, total cholesterol and hypertension.
